# MOSRS: An engineering multi-objective optimization through Einsteinian concept

**DOI:** 10.1371/journal.pone.0328005

**Published:** 2025-07-29

**Authors:** Vahid Goodarzimehr, João Luiz Junho Pereira, Nima Khodadadi

**Affiliations:** 1 Faculty of Civil Engineering and Architecture, Shahid Chamran University of Ahvaz, Ahvaz, Iran; 2 Manufacturing and Management Engineering Institute, Federal University of Itajubá, Itajubá, Minas Gerais, Brazil; 3 Department of Civil, Architectural and Environmental Engineering, University of Miami, Miami, Florida, United States of America; SR University, INDIA

## Abstract

Multi-objective optimization stands at the intersection of mathematics, engineering, and decision-making, and metaheuristics offer a promising avenue for tackling such challenges. The literature shows they are the best, and there is space for new algorithms to deliver Pareto Fronts (PFs) with more convergence and coverage at lower computational costs. This paper presents the Multi-objective Special Relativity Search (MOSRS) for the first time. It relies on principles inspired by the theory of special relativity physics, which iteratively refines solutions toward optimality and self-adapts its parameters using these laws. Unlike most algorithms in the literature today, the user sets only the number of iterations and particles (or population). To test the performance, MOSRS is applied to the most challenging test functions set (CEC 2009) and 21 real and constrained world problems, being compared with a total of eleven metaheuristics: NSGA-II, NSGA-III, MOEA/D, MOPSO, MOGWO, ARMOEA, TiGE2, CCMO, ToP, and AnD. Inverted Generational Distance, Spacing, Maximum Spread, and Hypervolume are used to identify the best algorithm. MOSRS was robust in finding the best PF in most studied problems. The source codes of the MOSRS algorithm are publicly available at https://nimakhodadadi.com/algorithms-%2B-codes.

## 1. Introduction

Structural optimization is crucial in various fields, such as engineering, architecture, and design, as it aims to find the best configuration of materials and shapes to achieve desired performance criteria while minimizing costs or other constraints. Metaheuristic algorithms play a significant role in this optimization process because they can efficiently explore large solution spaces and find near-optimal solutions in complex and often nonlinear problems. Particle Swarm Optimization (PSO) is one of the most popular algorithms developed to solve engineering problems, and it is inspired by the social behavior of bird flocking or fish schooling. It iteratively updates the position and velocity of particles in a multidimensional search space, guided by their own best-known position and the best-known position found by the entire swarm, aiming to efficiently explore and exploit the search space for optimal solutions [1].

Goodarzimehr et al. [2,3] presented a novel optimization technique inspired by the principles of Einstein’s theory of special relativity. The Special Relativity Search (SRS) algorithm leverages the concept of relativistic velocity addition to update particle positions and velocities in the search space. By incorporating relativistic effects, such as time dilation and length contraction, the SRS algorithm enhances the exploration-exploitation trade-off, enabling efficient and effective search for optimal solutions in complex optimization problems. Pereira et al. [4] introduced the Lichtenberg Algorithm (LA), a computational method inspired by the intricate branching patterns in electrical discharges. It simulates the growth of these patterns by iteratively applying probabilistic rules to determine the trajectory of discharges, yielding visually striking and complex fractal-like structures.

Scientists have developed various paradigms to address especially engineering problems by leveraging the rich advantages of metaheuristic methods, including artificial hummingbird algorithm [5], stochastic paint optimizer [6], starling murmuration optimizer [7], Advanced swarm algorithm [8], mountain gazelle optimizer [9], Improved chaos game optimization algorithm [10], Lichtenberg optimization algorithm [11], enhanced special relativity search algorithm [12], improved harmony search algorithm [13], modified adolescent identity search algorithm [14], greylag goose optimization [15], hybrid symmetry–PSO approach [16], electric eel foraging optimization [17], hippopotamus optimization algorithm [18], puma optimizer [19], among others. While all these methods are typically designed for optimizing single-objective problems, this study delves into investigating more challenging problems.

At its core, multi-objective optimization involves the exploration of a solution space to identify a set of solutions that are not dominated by any other solution, according to defined criteria. These solutions form what is known as the Pareto frontier or Pareto front, representing the trade-offs between conflicting objectives [20]. Each point on this frontier represents a feasible solution where improvement in one objective comes at the expense of another. The development of a metaheuristic algorithm for multi-objective optimization typically involves several key stages:

- Problem Understanding: The first step is thoroughly understanding the multi-objective optimization problem, including its objectives, constraints, and specific characteristics that may influence the solution space.
- Algorithm Design: Designing metaheuristic algorithms entails defining the search strategy, exploration, and exploitation mechanisms, as well as determining how solutions are represented and evaluated. This phase often draws inspiration from nature, such as evolutionary processes, swarm behavior, or physical phenomena.
- Implementation: Once the algorithm design is in place, the next step is implementing it in code. This involves translating the conceptual framework into a functional program capable of iteratively generating and evaluating solutions.
- Parameter Tuning: Metaheuristic algorithms typically have parameters influencing their behavior and performance. Fine-tuning these parameters through experimentation and optimization is crucial for achieving good results across different problem instances.
- Validation and Testing: Once implemented, the algorithm must undergo rigorous testing and validation to ensure its effectiveness and robustness. This involves benchmarking against known problem instances, comparing against existing algorithms, and assessing performance on real-world applications.
- Optimization and Enhancement: Continuous optimization and enhancement are essential for algorithm development. This may involve refining the search strategy, incorporating problem-specific knowledge, or adapting the algorithm to handle different problem types or constraints.

Building upon the abovementioned stages, scientists have developed various multi-objective methods to address engineering problems: Baril et al. [21] explored integrating Six Sigma methodologies with collaborative multi-objective optimization techniques to enhance product design processes. They highlight the synergies between these approaches and their potential to achieve simultaneous optimization of multiple conflicting objectives in complex engineering systems. Branke et al. [22] introduced a novel method that delves into strategies for providing direction and assistance to evolutionary algorithms when navigating complex multi-objective optimization problems. It explores various techniques to enhance convergence and diversity in the search process, ultimately aiding decision-makers in efficiently discussing and understanding the trade-offs among conflicting objectives. Coello et al. [23,24] developed different paradigms of multi-objective approaches for addressing engineering problems.

Cohon et al. [25] meticulously scrutinize diverse methodologies used in multi-objective programming, offering insights into their efficacy and practicality. This comprehensive analysis is a valuable resource for researchers and practitioners seeking to navigate the complexities of multi-objective optimization. Emmerich et al. [26] explore the foundational principles and evolutionary techniques employed in multi-objective optimization. This comprehensive guide equips readers with essential knowledge and practical insights for tackling complex optimization problems with conflicting objectives.

Fan et al. [27] investigate the optimal selection of heat pump technologies within micro-CHP systems, balancing multiple criteria for efficient energy utilization. This study offers valuable insights into enhancing the performance and sustainability of fuel cell-based micro-CHP systems through rigorous multi-objective optimization techniques. Fonseca et al. [28] comprehensively examine genetic algorithms tailored for multi-objective optimization problems. This paper delves into their formulation, discussion, and generalization, offering valuable insights into their applicability and efficiency across diverse optimization tasks. Gomes *et al.* have extensively investigated the application of metaheuristic algorithms for solving various complex multi-objective optimization problems [29,30].

Francisco et al. [31] explore the optimization of CRP Isogrid tubes through a multi-objective approach, leveraging the SunFlower optimization algorithm and metamodel-based techniques. This study offers valuable insights into enhancing composite materials’ design efficiency and performance in aerospace applications through multi-objective optimization methodologies. Jian et al. [32] introduce a novel evolutionary algorithm for optimizing many objectives, employing a reference-point-based nondominated sorting approach. This paper contributes to advancing multi-objective optimization techniques, offering a promising method for efficiently handling complex optimization problems with multiple conflicting objectives. Kumar et al. [33] present a comprehensive collection of real-world constrained multi-objective optimization problems and baseline performance results. This benchmark suite is a valuable resource for evaluating and comparing the efficacy of optimization algorithms in tackling complex real-world problems with multiple constraints and objectives.

Several other applications of multi-objective metaheuristics to handle structural problems can be found in literature, always showing that the most common and modern preference is to apply the metaheuristics to find all possible and non-dominated solutions first. This means the PF has more convergence and coverage at the same time, and then the decision maker analyses them and chooses the best one according to your preferences, but in general terms, all the solutions in the PF have initially equal importance [34–43].

This study will use the SRS algorithm, which has demonstrated excellent performance in its single-objective version, to construct a new multi-objective metaheuristic. The principles of special relativity inspire it, and it employs relativistic velocity addition to update particle positions and velocities in the search space efficiently. It also self-adapts its parameters using these laws. The user sets only the number of iterations and particles (or population) as stopping criteria, making it one of the few free specific -hyperparameters in the literature. The first step of the Multi-objective Special Relativity Search (MOSRS) algorithm is to define the objectives and constraints of the multi-objective optimization problem. These objectives may represent different performance metrics or criteria to be optimized simultaneously, and at least one must be conflictive. Next, initialize a population of solutions, or particles, within the search space. Then, during each iteration of the MOSRS algorithm, the positions and velocities of particles are updated based on their own best-known positions and the best-known positions found by the entire cluster (or population). These best positions are represented in the objective space as all current non-dominated solutions.

The relativistic velocity addition mechanism allows particles to explore the search space dynamically, considering the relative importance of objectives and constraints. Additionally, the MOSRS algorithm offers a unique advantage in handling dynamic environments or changing objectives. By continuously adapting particle velocities based on relativistic principles, the algorithm can dynamically adjust to changes in the optimization landscape, ensuring robust performance.

To validate the performance of the new MOSRS algorithm, the CEC 2009 [44] and 2021 [33] will be used. The former is considered the most challenging and used test functions in the literature [34,45,46], and MOSRS will be compared with the most used algorithms: NSGA-II, MOEA/D, MOPSO, and MOGWO. Inverted Generational Distance (IGS), Maximum Spread (MS), and Spacing (SP) are used to assess its performance because the True Pareto fronts (TPF) are available. Furthermore, 21 complex structural optimization problems from CEC 2021 are investigated. The proposed method is compared against seven recent methods: ARMOEA [47], TiGE2 [48], NSGA-III [32], CCMO [49], ToP [50], MOSFO [40], and AnD [51] using the Hypervolume (HV) metric, due to lack of available TPFs.

### 1.1. Related works

In recent structural optimization research, various bio-inspired and nature-inspired multi-objective algorithms have been introduced to address the complexity of truss design problems. The thermal exchange optimization was employed for multi-objective optimization, demonstrating improved convergence and solution diversity in truss structures [52]. Tejani et al. [53] applied a 2-archive multi-objective cuckoo search algorithm, effectively maintaining Pareto diversity while optimizing structural performance metrics. Similarly, the MOBBO algorithm, modeled after the behavior of brown bears, provided competitive results in constrained environments, indicating its potential as a viable alternative to classical methods.

Further enhancements have been explored through hybrid and archive-based mechanisms. The two-archive boosted MOHO algorithm was successfully applied to truss optimization, offering improved search capability and robustness compared to baseline models [54]. In parallel, the application of MOHO from a multi-objective perspective has also shown efficiency in generating well-distributed solutions. Multi-objective truss optimization has not been studied as extensively as its single-objective counterpart. To address this gap, the novel Multi-objective Lichtenberg Algorithm with Two Archives (MOLA-2arc) was developed by Panagant et al.. Its performance has been benchmarked against eight other multi-objective optimization algorithms, demonstrating its potential in handling complex structural design problems. A comprehensive analysis of many-objective metaheuristic methods emphasized the effectiveness of decomposition-based strategies and diverse search operators in high-dimensional objective spaces. The improved heat transfer search and its decomposition-based variant have also shown promise in handling constrained structural problems with conflicting objectives [55]. Multi-objective Generalized Normal Distribution Optimization (MOGNDO) algorithm was introduced in [56], an extension of the GNDO originally designed for single-objective problems. MOGNDO enhances the original approach by incorporating an archive to store non-dominated Pareto-optimal solutions and a new leader selection mechanism to guide the search. These improvements enable the algorithm to effectively balance convergence and diversity in solving multi-objective optimization tasks. These studies [52]–[56] form a strong foundation for advancing intelligent optimization techniques in structural engineering applications.

Complementing the above developments, several recent studies have focused on advancing many-objective optimization techniques tailored for complex engineering problems. Kalita et al. introduced the Many-Objective Multi-Verse Optimizer (MaOMVO), which integrates gravitational memory mechanisms to effectively handle convergence and diversity trade-offs in high-dimensional objective spaces [57]. Further, the MORKO algorithm, inspired by the Runge–Kutta numerical method, was proposed to tackle multi-domain optimization tasks and demonstrated strong generalization across varied benchmark functions [58]. Additional efforts include the development of the MaOGOA and the Many-Objective Whale Optimization Algorithm, both of which enhance solution distribution and convergence speed in large-scale, many-objective design problems [59] and [60]. Collectively, these approaches underscore the growing emphasis on scalable, general-purpose optimization frameworks capable of addressing the rising complexity in engineering design applications.

In parallel with these algorithmic advancements, efforts have also been directed toward enhancing the formulation and refinement of multi-objective optimization frameworks for structural and civil engineering applications. Abdel-Basset et al. proposed a balanced multi-objective optimization strategy based on improvement-guided reference points, effectively addressing convergence-stagnation issues commonly observed in multi-criteria [61]. More recently, MORIME, a robust multi-objective RIME framework, has been introduced specifically for efficient truss structure optimization, showing promising results in balancing structural efficiency and computational cost [62]. In domain-specific implementations, Mashru et al. [63] have applied various metaheuristics—ranging from many-objective approaches on complex spatial truss domes to thermal exchange optimization in structural systems—demonstrating their adaptability and effectiveness). In [64], the Multi-objective Stochastic Paint Optimizer (MOSPO) extends the original single-objective SPO by incorporating key features such as a fixed-sized external archive and a leader selection mechanism for effective multi-objective optimization. The proposed MOSPO was evaluated on ten benchmark functions (CEC-09) and eight engineering design problems, demonstrating superior accuracy and solution uniformity compared to other established methods.

Furthermore, application-oriented innovations such as the improved multi-objective particle swarm optimization algorithm for geotechnical projects highlight the growing relevance of these techniques in practical civil infrastructure scenarios [65]. These contributions collectively reinforce the trend toward more versatile and context-aware optimization models in contemporary engineering practice.

The paper is organized as follows: Section 2 presents the MOSRS. Section 3 describes the studied complex multi-objective problems, and Section 4 discusses their results and discussions. Section 5 concludes with the main highlights.

## 2. Multi-objective Special Relativity Search

MOSRS explores the Search Space with the population being charged particles, and to understand better how it works, we need to dive into physics. When moving through a magnetic field, a charged particle, such as an electron or a proton, experiences a force perpendicular to its velocity vector and the magnetic field lines. The Lorentz force law describes this force. For a positively charged particle, the force is in the direction of the field lines if the motion is perpendicular to the field lines, or it’s opposite if the motion is in the opposite direction to the field lines. For negatively charged particles, the direction of the force is reversed [2].

The Lorentz force describes the force experienced by a charged particle moving through an electromagnetic field. The equation gives it:


$$F_{j}=Q_{j}[E_{i}+v_{j}\times B_{i}]$$ (1)

$$B_{i}=\mu .Q_{i}.\frac{v_{i}}{{r_{ij}}^{3}}\vec{r}$$ (2)

where *F* is the Lorentz force vector, *Q* is the charge of the particle, *E* is the electric field vector, *v* is the velocity vector of the charged particle, *r* is the separate distance between two particles, $\overset{―}{r}$ is the unit vector, *μ* is the constant of the magnetic field, and *B* is the magnetic field vector.

This equation breaks down into two components:

a) The first component, *QE*, represents the force experienced by the charged particle due to the electric field. This force is directly proportional to the particle’s charge and the electric field’s strength.
b) The second component, *Q(v × B)*, represents the force experienced by the charged particle due to the magnetic field. It is proportional to both the charge of the particle and its velocity, as well as the strength and direction of the magnetic field.

In the magnetic field, the particles are moving at velocity *v*, but in the electric field, the particles don’t move, and their velocity is zero. Therefore, we neglect the electric term and rewrite Eq. (1) as follows:


$$F_{j}=Q_{j}v_{j}B_{i}$$ (3)

**Fig 1:**
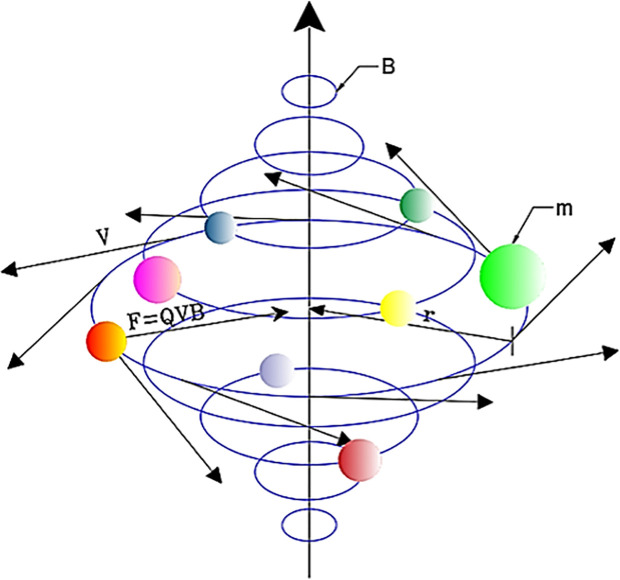
The direction of the magnetic force [1].

The direction of the magnetic force can be determined using the right-hand rule (Fig 1). If you point your thumb in the direction of the velocity of the charged particle and your fingers in the direction of the magnetic field, then the direction in which your palm faces give the direction of the force.

**Fig 2:**
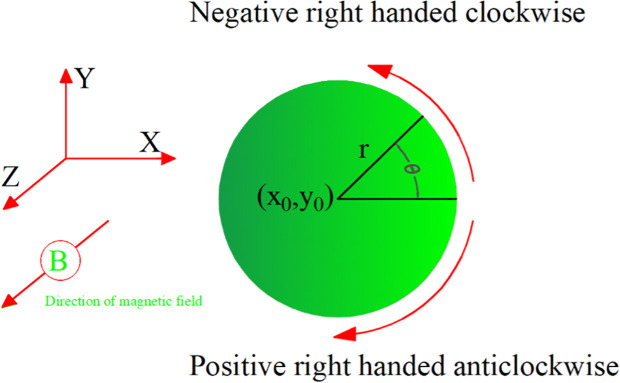
The radius of this circular motion.


As a result of this force, charged particles tend to move in circular or helical paths (spirals) if their initial motion has a component perpendicular to the magnetic field lines (Fig 2). The radius of this circular motion depends on the mass, charge, velocity, and strength of the magnetic field. The radius is defined using Eq. (4) and depicted in Fig 2.

$$r=\frac{mv}{QB}$$ (4)

The magnitude of the force experienced by the charged particle is proportional to its velocity and the strength of the magnetic field. Mathematically, this relationship is given by Eq. (3). This interaction between charged particles and magnetic fields is fundamental to various phenomena, such as the operation of electric motors, the behavior of particles in particle accelerators like cyclotrons, and the formation of auroras in Earth’s atmosphere.

The particles in a magnetic field have high speed and negligible mass. For this reason, Newtonian physics cannot mathematically represent the behavior of charged particles in a magnetic field. Special relativity physics is a good fit for simulating this kind of behavior. In relativity physics, the speed of particles is measured based on the speed of light, and by applying two significant phenomena, time dilation, and length contraction, the accuracy of equations is improved [66].

In this sense, in the 19th century, following the realization that the classical theory of electromagnetism was insufficient, there emerged a demand for a more comprehensive theory of relativity in physics. During this era, experimental findings regarding light propagation, particularly concerning the impact of an observer’s motion relative to the medium through which light traveled, challenged the prevailing beliefs of the time. To develop a description of light’s behavior consistent with these observations, the scientific community had to embrace the law proposed by Lorentz. This law established a connection between the coordinates of axes when in uniform relative motion, leading to what is now known as the Lorentz transformations. In essence, it can be stated that two fundamental principles govern the accurate transformation of coordinates in inertial frames according to relativity principles.

1. In every inertial frame, light moves uniformly in all directions at a consistent speed, denoted as ‘*c*’.
2. All reference frames, regardless of their initial state, are equally capable of accurately describing physical phenomena.

Applying the principles mentioned above allows for the initial derivation of Lorentz transformations. Thus, considering two orthogonal coordinate frames, *E*_*1*_ and *E*_*2*_, moving with a constant relative speed *U* along their *X* axis, denoted as (*X*), the transformation between the coordinates of an event in the first frame, represented as *x*_*1*_, *y*_*1*_, *z*_*1*_, *t*_*1*_, and the coordinates of the same event in the second frame, denoted as *x*_*2*_, *y*_*2*_, *z*_*2*_, and *t*_*2*_ can be expressed through Lorentz transformations. These transformations describe the relation between the coordinates of the event when transitioning from one frame to another and are outlined as follows.


$$t_{2}=\frac{t_{1}-\frac{v}{c^{2}}x_{1}}{\sqrt{1-(\frac{v}{c}{)}^{2}}}\Rightarrow t_{1}-(\frac{v}{c^{2}})x_{1}=t_{2}\sqrt{1-(\frac{v}{c}{)}^{2}}\Rightarrow t_{1}=(\frac{v}{c^{2}})x_{1}+t_{2}\sqrt{1-(\frac{v}{c}{)}^{2}}$$ (5)

$$x_{2}=\frac{x_{1}-vt}{\sqrt{1-(\frac{v}{c}{)}^{2}}}\Rightarrow x_{1}-vt_{1}=x_{2}\sqrt{1-(\frac{v}{c}{)}^{2}}\Rightarrow x_{1}=vt_{1}+x_{2}\sqrt{1-(\frac{v}{c}{)}^{2}}$$ (6)

$$x_{1}=(\frac{v}{c}{)}^{2}x_{1}+vt_{2}\sqrt{1-(\frac{v}{c}{)}^{2}}+x_{2}\sqrt{1-(\frac{v}{c}{)}^{2}}$$ (7)

$$v=\omega_{n}r=\frac{QB}{m}r=\mu \frac{Q_{i}Q_{j}}{m{r_{ij}}^{3}}v_{j}r_{ij}=\mu \frac{Q_{i}Q_{j}}{m{r_{ij}}^{2}}v_{j}$$ (8)

Eq. (7) represents a pivotal step in the MOSRS algorithm. It incorporates both the initial and destination positions, velocity, and direction. It’s evident that the equation takes on a distinct form, mainly due to coordinates being measured relative to the speed of light.

### 2.1. Mathematical simulation of the MOSRS algorithm

Expanding the single-objective framework of the Special Relativity Search (SRS) algorithm to tackle multiple conflicting objectives results in a multi-objective version. This is done by integrating ideas of multi-objective optimization like Pareto dominance and preservation of diversity. In multi-objective optimization, multiple objectives are optimized concurrently rather than using a single fitness function. The main ideas to present are:

- Pareto dominance: A solution y_1_ dominates another solution y_2_ when y_1_ is equal to or better than y_2_ in all objectives and superior in at least one.
- Pareto front: A set of non-dominated solutions that are considered optimal.
- Diversity maintenance: Ensures that there are diverse solutions along the Pareto front.

Initially, the multi-objective process commences with a randomly created population of particles, each symbolizing a possible solution. The particles will be evaluated based on multiple objective functions. The particles are organized by Pareto dominance to detect fronts that are not dominated. Every particle is ranked according to its present location, and the vector of the best points (ideal point) and the worst (nadir point) is used to calculate the particle’s charge.

The velocity and position of particles need to be updated, considering time dilation and length contraction. The updates must be guided by both the current position and the non-dominated solutions. Particles are selected for the next generation based on rank and diversity measures. Selection and replacement are performed as follows:

- Combine the current population *X* and the new population *X′* (generated from the updated positions) into a combined population *X_c_ = X*∪*X*′.
- Perform non-dominated sorting on *X*_*c*_ and select the top *N* particles based on Pareto fronts and diversity measures to form the next generation.

Repeat the evaluation, sorting, updating, and selection until a stopping criterion is met. The final set of non-dominated solutions forms the Pareto front, representing the optimal trade-offs among the objectives.

A magnetic field is conceptualized as the feasible search space, where all optimal solutions within this space are deemed acceptable. The boundaries of the search space are defined as side constraints. The particles exert forces on each other, moving towards optimal solutions. The optimization process begins with a random solution. Subsequently, the search process continues until the stop criterion is met. In the MOSRS algorithm, the stop criterion is determined by two different loops. In the first loop, the stop criterion is the population size, while in the second loop, it is the maximum iteration. The initial population is defined using Eq. (9). According to Eq. (10), the fitness of the optimal solutions is evaluated in each iteration.


$$X_{ij}=[\begin{array}{cccccc} X_{11} & X_{12} & \cdots & X_{1j} & \cdots & X_{1n}\\ X_{21} & X_{22} & \cdots & X_{2j} & \cdots & X_{2n}\\ \vdots & \vdots & \vdots & \vdots & \vdots & \vdots \\ X_{i1} & X_{i2} & \cdots & X_{ij} & \cdots & X_{in}\\ \vdots & \vdots & \vdots & \vdots & \vdots & \vdots \\ X_{P1} & X_{P2} & \cdots & X_{Pj} & \cdots & X_{Pn} \end{array}]$$ (9)

$$x_{ij}^{new}=LB+rand\times (UB-LB)$$ (10)

where *x*_*ij*_ is the vector of possible optimal solutions, *LB* and *UB* are the lower and upper bound, respectively. *rand* is a stochastic number between [0,1]. The distance and charge of particles are calculated using Eq. (11) and Eq. (12), respectively.


$$r_{ij}^{t}=\frac{norm(X_{i}^{t}-X_{j}^{t})}{X_{i}^{t}+X_{j}^{t}}$$ (11)

$$Q_{ik}^{t}=fit_{ik}^{t}-{worst}_{k}^{t}\;\;{best}_{k}^{t}-{worst}_{k}^{t}$$ (12)

where *fit* is the objective function value for the *k*^*th*^ objective on the iteration *i*^*th*^ and *best* and *worst* are the best and worst for these objectives according to the current Pareto front. That is, the nadir and ideal vector points. Eq. (13) is applied to determine the position of the particle. This equation depends on cyclotron frequency. The cyclotron frequency is calculated using Eq. (14).


$$x_{2}=x_{0}+r\times \sin(\omega_{n}t)$$ (13)

$$\omega_{n}=\mu \frac{Q_{i}Q_{j}v_{j}}{mr_{ij}^{3}}$$ (14)

Then, the main equation of step length of the MOSRS algorithm is defined using the relativistic Eq. (15).


$$x_{new}^{t}=\beta^{2}x_{old}^{t}+\left[\mu \frac{Q_{i}Q_{j}}{m{r_{ij}}^{2}}v_{j}\right]\sqrt{1-\beta^{2}}+x_{2}\sqrt{1-\beta^{2}}$$ (15)

where *β* represents the relativistic parameter, indicating the ratio of the speed of particles to the speed of light (approximately 3 × 10^8^ meters per second). In the standard SRS, *β* is modeled using a stochastic operator and assigned exact values here. Note that MOSRS has self-adaptative parameters according to the fact, and only several iterations and particles rule its routine.

**Fig 3:**
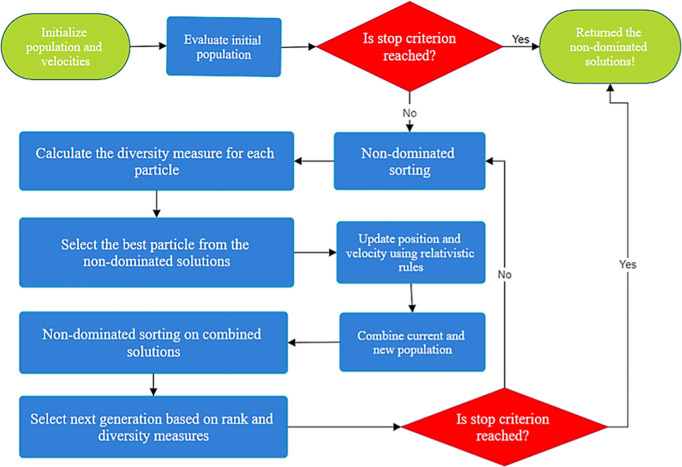
Flowchart of MOSRS algorithm.

Each particle in the search space generates a vector of solutions in the objective space. At each iteration, the new solutions are integrated with the current Pareto front and analyzed by the Pareto dominance relationship that excludes the dominated solutions. Our method keeps only *Ns* solutions in the Pareto front to improve computational cost. The criteria to keep or not them is how close one is to another using Euclidian distance. Also, our proposed method is equipped with the “death penalty” function, which penalizes solutions that violate equality or inequality constraints. The flowchart of the Multi-Objective Special Relativity Search (MOSRS) algorithm is presented in Fig 3 to clarify the main steps and decision mechanisms. Table 1 brings its pseudocode.

**Table 1:** MOSRS’s pseudocode.

Set the search space and objective function – upper and lower bounds and *J* Set the population and iteration numbers – *Pop* and *N*_*iter*_ (common to all MH) Start: Initialize population and velocities. Evaluate Initial Population: Calculate fitness values for each particle. **While** not Stopping Criterion: Non-dominated Sorting: Sort particles into Pareto fronts. Calculate Diversity Measure: Compute crowding distance or another diversity metric. Update Particles: Select Best Particle: From non-dominated solutions. Update Position and Velocity: Using relativistic rules. Evaluate New Positions: Calculate fitness values for new positions. Combine Populations: Current and new particles. Non-dominated Sorting on Combined Population: Sort into Pareto fronts. Select Next Generation: Based on rank and diversity measures. **End While**: Check stopping criterion. Output Pareto Front: Return the set of non-dominated solutions

### 3. Test problems

MOSRS will be tested through two CEC test functions: CEC 2009 for complex and general multi-objective optimization problems and CEC 2021 for real-world design problems.

### 3.1. CEC 2009 test functions

This test suite was proposed by Zhang et al. [44] and is considered by many authors as the most complex test functions for multi-objective problems until today; those used here are presented in Table 2. MOSRS will be compared against the most used algorithms in literature today in these functions. They and their recommended hyperparameters by other studies are in Table 3. Note that MOSRS does not have specific hyperparameters.

**Table 2:** Hardest general multi-objective problems.

Name	Mathematical formulation
UF1	$\begin{array}{c} f_{1}(x)=x_{1}+\frac{2}{\left|j_{1}\right|}{\sum_{j\in J_{1}}\left[x_{j}-\sin(6\pi x_{1}+\frac{j\pi }{n})\right]}^{2},f_{2}(x)=1-\sqrt{x}+\frac{2}{\left|j_{2}\right|}{\sum_{j\in J_{2}}\left[x_{j}-\sin(6\pi x_{1}+\frac{j\pi }{n})\right]}^{2} \end{array}$ $J_{1}=\;\left\{j|j\;is\;odd\;and\;2\leq j\leq n\right\},\;\;J_{2}=\;\left\{j|j\;is\;even\;and\;2\leq j\leq n\right\}\;\;$
UF2	$\begin{array}{c} f_{1}(x)=x_{1}+\frac{2}{\left|j_{1}\right|}\sum_{j\in J_{1}}y_{j}^{2},f_{2}(x)=1-\sqrt{x}+\frac{2}{\left|j_{2}\right|}{\sum_{j\in J_{2}}y_{j}}^{2} \end{array}$ $J_{1}and\;J_{2}are\;the\;same\;of\;UF1$
UF3	$\begin{array}{c} f_{1}(x)=x_{1}+\frac{2}{\left|j_{1}\right|}(4\sum_{j\in J_{1}}y_{j}^{2}-2\prod_{j\in j_{1}}\cos(\frac{20y_{j}\pi }{\sqrt{j}})+2),f_{2}(x)=\sqrt{x_{1}}+\frac{2}{\left|j_{2}\right|}(4\sum_{j\in J_{1}}y_{j}^{2}-2\prod_{j\in j_{2}}\cos(\frac{20y_{j}\pi }{\sqrt{j}})+2) \end{array}$ $J_{1}and\;J_{2}are\;the\;same\;of\;UF1;y_{j}=x_{j}-x_{1}^{0.5\left(1+\frac{3(j-2)}{n-2}\right)},\;j=2,\;3,\;\ldots ,\;n\;$
UF4	$\begin{array}{c} f_{1}(x)=x_{1}+\frac{2}{\left|j_{1}\right|}\sum_{j\in J_{1}}h(y_{j}),f_{2}(x)=1-x_{2}+\frac{2}{\left|j_{2}\right|}\sum_{j\in J_{2}}h(y_{j})\;\; \end{array}$ $J_{1}and\;J_{2}are\;the\;same\;of\;UF1,\;y_{j}=x_{j}-\sin\left(6\pi x1\;+\frac{j\pi }{n}\right),\;j=2,3,\ldots ,n,h(t)=\frac{\left|t\right|}{1+e^{2|t|}}$
UF5	$\begin{array}{c} f_{1}(x)=x_{1}+(\frac{1}{2N}+\varepsilon )|\sin(2N\pi x_{1})|+\frac{2}{\left|J_{1}\right|}\sum_{j\in J_{1}}h(y_{i}) \end{array}$ $\begin{array}{c} f_{2}(x)=1-x_{1}+(\frac{1}{2N}+\varepsilon )|\sin(2N\pi x_{1})|+\frac{2}{\left|J_{2}\right|}\sum_{j\in J_{2}}h(y_{i}) \end{array}$ $J_{1}and\;J_{2}are\;the\;same\;of\;UF1,\varepsilon >0,\;y_{j}=x_{j}-\sin\left(6\pi x1\;+\frac{j\pi }{n}\right),\;j=2,3,\ldots ,n,h(t)=2t^{2}-\cos(4\pi t)+1\;\;$
UF6	$\begin{array}{c} f_{1}(x)=x_{1}+\max\left\{0,2(\frac{1}{2N}+\varepsilon )\sin(2N\pi x_{1})\right\}+\frac{2}{\left|J_{1}\right|}\left(4\sum_{j\in j_{1}}y_{j}^{2}-2\prod_{j\in j_{1}}\cos(\frac{20y_{j}\pi }{\sqrt{j}})+2\right) \end{array}$ $\begin{array}{c} f_{2}(x)=1-x_{1}+\max\left\{0,2(\frac{1}{2N}+\varepsilon )\sin(2N\pi x_{1})\right\}+\frac{2}{\left|J_{2}\right|}\left(4\sum_{j\in j_{1}}y_{j}^{2}-2\prod_{j\in j_{2}}\cos(\frac{20y_{j}\pi }{\sqrt{j}})+2\right) \end{array}$ $J_{1}and\;J_{2}are\;the\;same\;of\;UF1,\varepsilon >0,,\;y_{j}=x_{j}-\sin\left(6\pi x1\;+\frac{j\pi }{n}\right),\;j=2,3,\ldots ,n\;\;$
UF7	$\begin{array}{c} f_{1}(x)=\sqrt[{5}]{x_{1}}+\frac{2}{\left|J_{1}\right|}\sum_{j\in J_{1}}y_{j}^{2},f_{2}(x)=1-\sqrt[{5}]{x_{1}}+\frac{2}{\left|J_{2}\right|}\sum_{j\in J_{2}}y_{j}^{2} \end{array}$ $J_{1}and\;J_{2}are\;the\;same\;of\;UF1,\varepsilon >0,,\;y_{j}=x_{j}-\sin\left(6\pi x1\;+\frac{j\pi }{n}\right),\;j=2,3,\ldots ,n$
UF8	$\begin{array}{c} f_{1}(x)=\cos(0,5x_{1}\pi )\cos(0,5x_{2}\pi )+\frac{2}{\left|j_{1}\right|}\sum_{j\in J_{1}}\left[x_{j}-2x_{2}\sin(2\pi x_{1}+\frac{j\pi }{n}{)}^{2}\right] \end{array}$ $\begin{array}{c} f_{2}(x)=\cos(0,5x_{1}\pi )\sin(0,5x_{2}\pi )+\frac{2}{\left|j_{1}\right|}\sum_{j\in J_{1}}\left[x_{j}-2x_{2}\sin(2\pi x_{1}+\frac{j\pi }{n}{)}^{2}\right] \end{array}$ $\begin{array}{c} f_{3}(x)=\sin(0,5x_{1}\pi )+\frac{2}{\left|j_{3}\right|}\sum_{j\in J_{3}}\left[x_{j}-2x_{2}\sin(2\pi x_{1}+\frac{j\pi }{n}{)}^{2}\right]\; \end{array}$ $\begin{array}{cc} \;where\; & J_{1}=\;\left\{j|3\leq j\leq n,and\;j-1\;is\;a\;multiplication\;of\;3\right\},\;\;J_{2}=\;\left\{j|3\;\leq j\leq n,and\;j-2\;is\;a\;multiplication\;of\;3\right\},\\ & J_{3}=\;\left\{j|3\;\leq j\leq n,and\;j\;is\;a\;multiplication\;of\;3\right\} \end{array}$
UF9	$\begin{array}{c} f_{1}(x)=0.5\left[\max\left\{0,(1+\in )(1-4(2x_{1}-1{)}^{2}\right\}+2x_{1}\right]x_{2}+\frac{2}{\left|j_{1}\right|}\sum_{j\in j_{1}}\left(x_{j}-2x_{2}\sin(2\pi x_{1}+\frac{j\pi }{n}{)}^{2}\right) \end{array}$ $\begin{array}{c} f_{2}(x)=0.5\left[\max\left\{0,(1+\in )(1-4(2x_{1}-1{)}^{2}\right\}+2x_{1}\right]x_{2}+\frac{2}{\left|j_{2}\right|}\sum_{j\in j_{2}}\left(x_{j}-2x_{2}\sin(2\pi x_{1}+\frac{j\pi }{n}{)}^{2}\right) \end{array}$ $\begin{array}{c} f_{3}(x)=1-x_{2}+\frac{2}{\left|j_{3}\right|}\sum_{j\in j_{3}}\left(x_{j}-2x_{2}\sin(2\pi x_{1}+\frac{j\pi }{n}{)}^{2}\right) \end{array}$ $\begin{array}{cc} \;\;where\; & J_{1}=\;\left\{j|3\leq j\leq n,and\;j-1\;is\;a\;multiplication\;of\;3\right\},\;J_{2}=\;\left\{j|3\;\leq j\leq n,and\;j-2\;is\;a\;multiplication\;of\;3\right\},\\ & J_{3}=\;\left\{j|3\;\leq j\leq n,and\;j\;is\;a\;multiplication\;of\;3\right\},\;\in \;=0.1; \end{array}$
UF10	$\begin{array}{c} f_{1}(x)=\cos(0,5x_{1}\pi )\cos(0,5x_{2}\pi )+\frac{2}{\left|j_{1}\right|}\sum_{j\in J_{1}}\left[4y_{j}^{2}-\cos(8\pi y_{j})+1\right] \end{array}$ $\begin{array}{c} f_{2}(x)=\cos(0,5x_{1}\pi )\cos(0,5x_{2}\pi )+\frac{2}{\left|j_{2}\right|}\sum_{j\in J_{1}}\left[4y_{j}^{2}-\cos(8\pi y_{j})+1\right] \end{array}$ $\begin{array}{c} f_{3}(x)=\sin(0,5x_{1}\pi )+\frac{2}{\left|j_{3}\right|}\sum_{j\in J_{3}}\left[4y_{j}^{2}-\cos(8\pi y_{j})+1\right] \end{array}$ $\begin{array}{cc} \;where\; & J_{1}=\;\left\{j|3\leq j\leq n,and\;j-1\;is\;a\;multiplication\;of\;3\right\},\;J_{2}=\;\left\{j|3\;\leq j\leq n,and\;j-2\;is\;a\;multiplication\;of\;3\right\},\\ & J_{3}=\;\left\{j|3\;\leq j\leq n,and\;j\;is\;a\;multiplication\;of\;3\right\} \end{array}$

**Table 3:** Commonly used metaheuristics Hyperparameters.

General		
	Number of iterations	3000
	Number of search agents	100
**Algorithm**	**Specifics**	**Value**
MOSRS	–	–
MOPSO	The cognitive coefficient and social coefficient	2.05
	The inertia weight	0.9
	The velocity of particle movement	0.5
	leader selection pressure parameter (*β*)	4
	number of grids per dimension (*nGrid*)	10
NSGA-II	Crossover rate	0.8
	Mutation rate	0.1
MOGWO	grid inflation parameter (*α*)	0.1
	leader selection pressure parameter (*β*)	4
	number of grids per dimension (*nGrid*)	10
MOEA/D	subproblems (*N*)	100
	neighbors (*T*)	10
	maximal copies of a new child in the update (*n*_*r*_)	1
	probability of selecting parents from the neighborhood (*δ*)	0.9
	mutation rate (*F*)	0.5
	distribution index (*η*)	30

Three metrics, inverted generational distance (IGD), maximum spread (MS), and spacing (SP), will be used to evaluate the performance of these algorithms.

**Inverted Generational Distance (IGD)**: This method is a performance metric in multi-objective optimization algorithms used to evaluate the quality of approximate sets generated by these algorithms. This method calculates the distance between the points of the approximate set and the points of an ideal reference set. Specifically, IGD measures the average distance from each point in the reference set to the nearest point in the approximate set. A lower IGD value indicates that the approximate set is closer to the reference set, thereby better evaluating the performance of the optimization algorithm. Due to its high accuracy and reliability, IGD is very common in comparing and analyzing the performance of different multi-objective optimization algorithms. The IGD quantifies how much the metaheuristic approaches the Pareto front, measuring its ability to converge, and it is in Eq (16):


IGD=\raise0.7ex\({\sqrt {\sum\limits_{I = 1nt(di′)2}\)}/∑I=1nt(di′)2n\nulldelimiterspace\lower0.7ex\(n\)} (16)

where *nt* is the number of true Pareto optimal solutions; **d’*_*i*_* indicates the Euclidean distance between the *i*-*th* true Pareto optimal solution and the closest Pareto optimal solution obtained in the reference set.

**Maximum Spread (MS)**: The MS examines and measures the extent of coverage and dispersion of points in the objective space. Specifically, Maximum Spread calculates the distance between the farthest points in the approximate set. A higher value of this criterion indicates that the approximate set has greater diversity and dispersion in the objective space. Using Maximum Spread is particularly useful when maintaining diversity in the population of optimal points, as this diversity can help find better solutions and avoid local optimal points. This method serves as a key tool in analyzing the performance of multi-objective optimization algorithms, assisting researchers to identify and develop algorithms capable of producing diverse and well-dispersed sets.

**Spacing (SP)**: The SP checks the distances between the points in the approximate set to determine whether the solutions are uniformly distributed across the Pareto front. Specifically, SP calculates the standard deviation of the consecutive distances between points. A lower SP value indicates a more uniform and orderly distribution of points, which means better diversity and more complete coverage of the objective space. The SP metric is particularly important in multi-objective optimization problems because the uniform distribution of points can help identify and maintain diversity in the set of optimal solutions, preventing excessive clustering of solutions in specific areas.

The SP and the MS measure the coverage, and they are given by Eqs 17 and18, respectively:


$$SP=\sqrt{\frac{1}{n-1}\sum_{I=1}^{n}{\left(\bar{d}-d_{i}\right)}^{2}}$$ (17)

where $\overset{―}{d}$ is the average of all *d*_*i*_; *n* is the number of Pareto optimal solutions obtained; and


$$d_{i}=\left(\left|f_{1}^{\;i}\left(\vec{x}\right)-f_{1}^{\;j}\left(\vec{x}\right)\right|+\left|f_{2}^{\;i}\left(\vec{x}\right)-f_{2}^{\;j}\left(\vec{x}\right)\right|\right)\;for\;all\;i,\;j\;=\;1,\;2,\;3,\;\ldots ,\;n.$$

$$MS=\sqrt{\sum_{i=1}^{o}\max(d(a_{i},b_{i}))}$$ (18)

where *d* is a function to calculate the Euclidean distance; *a*_i_ is the maximum value in the *i*-*th* objective; *b*_i_ is the minimum in the *i*-*th* objective; and *o* is the number of objectives.

The lower values of IGD and SP represent better results, while for MS is the opposite. All the algorithms will be run 30 times to obtain the average and standard deviation (SD).

### 3.2. Real-world optimization problems

The proposed method will also be tested on 21 complex mechanically constrained multi-objective design problems. The problem’s name, the variables number (*N*_*v*_), the objectives number (*N*_*o*_), the inequality constraints number (*N*_*i*_), and the equality constraints number (*N*_*e*_) are in Table 4. More and more information can be found in the references after the name and in Kumar et al. [33]. We can see that the Speed Reducer project and the Car Inside impact are the problems with more design variables (7). The more dimensional search space is, the harder it is for metaheuristics.

**Table 4:** Real design multi-objective problems (Adapted from [33]).

Problem	Name	*N*_*v*_	*N*_*o*_	*N*_*i*_	*N*_*e*_
RCM01	Pressure Vessel [67]	4	2	2	2
RCM02	Vibration Platform [68]	5	2	5	0
RCM03	Two Bar Truss [69]	3	2	3	0
RCM04	Welded Beam [70]	4	2	4	0
RCM05	Disc Brake [71]	4	2	4	0
RCM06	Speed Reducer [72]	7	2	11	0
RCM07	Gear Train [73]	4	2	1	0
RCM08	Car Side Impact [32]	7	3	9	0
RCM09	Four Bar Plane Truss [74]	4	2	0	0
RCM10	Two Bar Plane Truss [33]	2	2	2	0
RCM11	Water Resources Management [33]	5	3	7	0
RCM12	Simply Supported beam [75]	2	4	1	0
RCM13	Gear Box Design [33]	3	7	11	0
RCM14	Multiple Disk Clutch Brake [76]	2	5	8	0
RCM15	Spring [77]	2	3	8	0
RCM16	Cantilever Beam [78]	2	2	2	0
RCM17	Bulk Carrier [79]	3	6	9	0
RCM18	Front Rail [80]	2	3	3	0
RCM19	Multi-product Batch Plant [81]	3	10	10	0
RCM20	Hydro-static Thrust Bearing [82]	2	4	7	0
RCM21	Crash Energy for High-Speed Train [83]	2	6	4	0

Otherwise, the most complex objective spaces in the Multi-product Batch Plant, Gear Box Design, Crash Energy for High-Speed Train, and Bulk Carrier designs represent a metaheuristics challenge. But of course, just analyzing the number of the dimensions. We will apply MOSRS to address these optimization problems and compare its performance with other recent algorithms, such as ARMOEA, TiGE 2, NSGA-III, CCMO, ToP, MOSFO, and AnD.

Our proposed method does not have specific parameters, only a number of particles and iterations. The other algorithm parameters are detailed in their respective publications; we will adopt the recommendations of Kamur *et al.* [33] and Pereira & Gomes [40]. For a fair comparison, we will use the same number of iterations and population for all the metaheuristics, following what was proposed by Kumar et al. [33]: for *N*_*v*_ values of 2, 3, 4, and 5, the population sizes will be set to 80, 105, 143, and 212, respectively. If *N*_*v*_ is less than or equal to 10, the number of iterations will be fixed at 2500; if *N*_*v*_ is greater than 10, the number of iterations will be set to 10000. As with other real problems, these multi-objective problems do not have a clearly defined and true Pareto front. Therefore, we will use the Hypervolume (HV) metric to compare the algorithms. It measures the volume of the objective space dominated by the Pareto front and the Nadir points. The same found by Kumar et al. [33] will be used and presented in their paper for a fair comparison. Maximizing the hypervolume helps maintain a diverse and extensive Pareto front.

**Fig 4:**
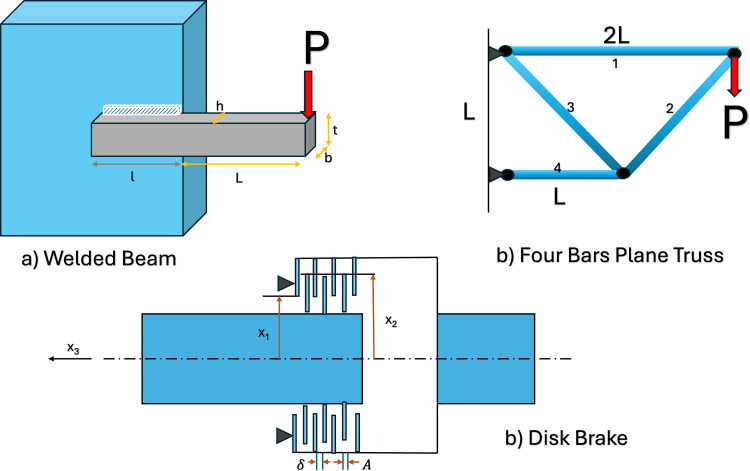
Schematic of some engineering problems [34].

This metric utilizes a reference vector, denoted as *r*, to calculate the space between the reference vector and the non-dominated solutions constituting the Pareto front. Typically, the reference vector is the nadir point, and the objective space is normalized using this vector. A higher HV indicates greater convergence and coverage of the Pareto front simultaneously. The reference vectors for CEC 2021 multi-objective problems have been outlined in Kamur et al. [33]. Also, Fig 4 represents some of the problems with *N*_*v *_= 4.

## 4. Results and discussions

### 4.1. CEC 2009

**Fig 5:**
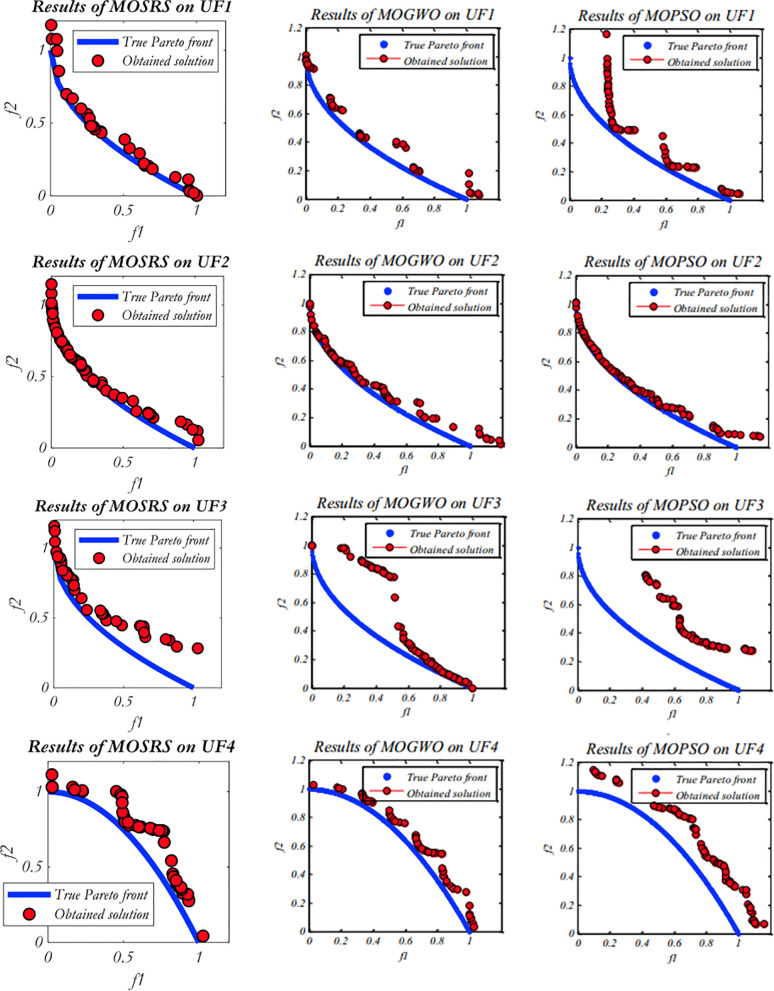
CEC 2009 rounds for some algorithms, highlighting MOSRS (UF1:UF4).

**Fig 6:**
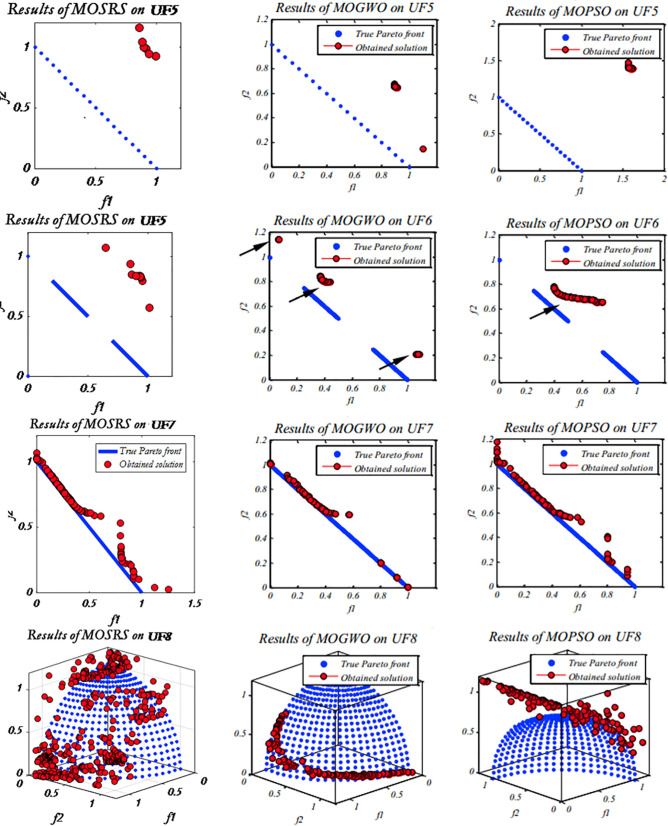
CEC 2009 rounds for some algorithms, highlighting MOSRS (UF5:UF8).

**Fig 7:**
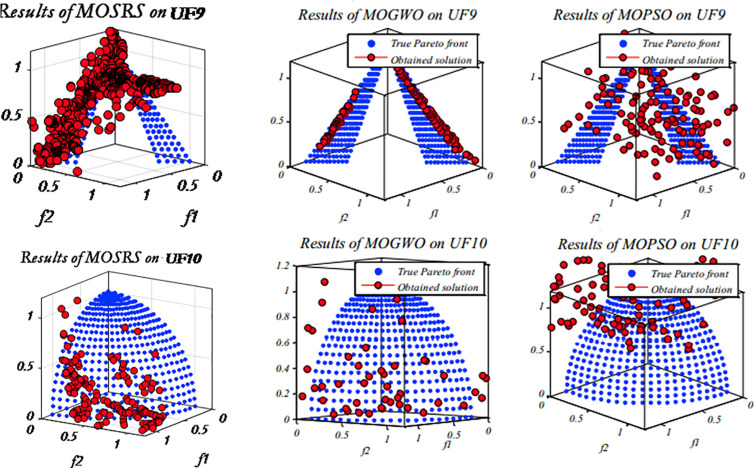
CEC 2009 rounds for some algorithms, highlighting MOSRS (UF9-UF10).

Five multi-objective metaheuristics were compared on the CEC2009 functions: MOSRS, MOGWO, MOPSO, NSGA-II, and MOEA/D. The computer used was a DELL core i7 with 16GB RAM SSD and 1 TB HD, and all algorithms are implemented in MATLAB. Figs 5–7 show the Pareto fronts for some of the executions for MOSRS, MOGWO, and MOPSO. It shows the complexity of the testing functions and the difficulties of the algorithms. Tables 5–7 show the mean results after 30 independent runs for all algorithms for statistical analysis of the metrics used in this study: IGD, SP, and MS, respectively.

**Table 5:** IGD results.

UF	Algorithm	Average	SD	UF	Average	SD
1	**MOSRS**	**0.10794**	0.00537	2	0.06911	0.00945
	MOGWO	0.11442	0.01954		0.05825	0.00739
	MOPSO	0.13700	0.04407		0.06040	0.02762
	NSGA-II	0.18640	0.01911		**0.04492**	0.00772
	MOEA/D	0.18710	0.05070		0.12230	0.01070
3	**MOSRS**	0.26591	0.01583	4	**0.05623**	0.00349
	MOGWO	**0.25569**	0.08070		0.05867	0.00048
	MOPSO	0.31399	0.04473		0.13504	0.00739
	NSGA-II	0.27400	0.03691		0.09661	0.01073
	MOEA/D	0.28865	0.01592		0.06810	0.00210
5	**MOSRS**	1.18312	0.14066	6	**0.24097**	0.06561
	MOGWO	**0.79707**	0.37857		0.27937	0.10448
	MOPSO	2.20237	0.55304		0.64752	0.26612
	NSGA-II	1.37961	0.22912		0.51132	0.13572
	MOEA/D	1.29145	0.13489		0.68812	0.05533
7	**MOSRS**	**0.13604**	0.01097	8	1.93853	0.02361
	MOGWO	0.16036	0.13911		2.05777	1.14552
	MOPSO	0.35395	0.20442		**0.53671**	0.18257
	NSGA-II	0.24872	0.09733		1.47756	0.37454
	MOEA/D	0.45520	0.18980		–	–
9	**MOSRS**	**0.18548**	0.12560	10	2.20242	0.30028
	MOGWO	0.19174	0.09250		3.59453	3.48829
	MOPSO	0.48850	0.14449		**1.63719**	0.29879
	NSGA-II	0.24162	0.15545		4.64931	1.10352
	MOEA/D	–	–		–	–

**Table 6:** SP results of algorithms in the CEC 2009 test functions.

UF	Algorithm	Average	SD	UF	Average	SD
01	**MOSRS**	0.01681	0.00441	2	0.01066	0.00974
	MOGWO	0.01237	0.01462		0.01108	0.00362
	MOPSO	0.00898	0.00247		**0.00829**	0.00168
	NSGA-II	0.01072	0.00371		0.01122	0.00902
	MOEA/D	**0.00380**	0.00150		0.00880	0.00080
3	**MOSRS**	0.00465	0.00416	4	0.00918	0.00140
	MOGWO	0.04590	0.01453		0.00969	0.00390
	MOPSO	**0.00699**	0.00170		**0.00666**	0.00091
	NSGA-II	0.02214	0.01471		0.00842	0.00163
	MOEA/D	0.02680	0.02064		0.00730	0.00060
5	**MOSRS**	0.07296	0.03367	6	**0.03883**	0.01899
	MOGWO	0.15231	0.16247		0.01446	0.01246
	MOPSO	0.00479	0.00408		0.02084	0.03258
	NSGA-II	0.02346	0.01753		0.02692	0.01701
	MOEA/D	**0.00278**	0.00553		0.00630	0.01267
7	**MOSRS**	0.00780	0.00683	8	0.01264	0.03475
	MOGWO	0.00824	0.00856		**0.00687**	0.00474
	MOPSO	0.00670	0.00285		0.02682	0.00827
	NSGA-II	0.00433	0.00340		0.02635	0.01489
	MOEA/D	**0.00540**	0.00300		–	–
9	**MOSRS**	0.05897	0.00947	10	0.03581	0.02264
	MOGWO	**0.01743**	0.00633		0.02523	0.01500
	MOPSO	0.02343	0.00405		**0.01994**	0.00348
	NSGA-II	0.03001	0.01007		0.05354	0.01826
	MOEA/D	–	–		–	–

**Table 7:** MS results of algorithms in the CEC 2009 test functions.

UF	Algorithm	Average	SD	UF	Average	SD
1	**MOSRS**	**0.99448**	0.19387	2	**0.96413**	0.01867
	MOGWO	0.92680	0.06884		0.90972	0.02867
	MOPSO	0.64538	0.19292		0.91205	0.02560
	NSGA-II	0.81776	0.25771		0.89092	0.02518
	MOEA/D	0.51770	0.16610		0.87200	0.00560
3	**MOSRS**	0.94421	0.00000	4	**0.98501**	0.01576
	MOGWO	**0.94982**	0.08777		0.94242	0.00093
	MOPSO	0.61030	0.10575		0.81275	0.01367
	NSGA-II	0.89571	0.11651		0.91372	0.05922
	MOEA/D	0.23994	0.12129		0.88320	0.01810
5	**MOSRS**	**0.68091**	0.07427	6	0.68945	0.17045
	MOGWO	0.39503	0.17494		**0.67360**	0.12323
	MOPSO	0.27926	0.09575		0.27435	0.11285
	NSGA-II	0.51391	0.86173		0.34615	0.14321
	MOEA/D	0.29215	0.03470		0.09677	0.20715
7	**MOSRS**	**0.96587**	0.28478	8	**0.65187**	0.28716
	MOGWO	0.80126	0.30865		0.44573	0.18574
	MOPSO	0.42928	0.27553		0.50810	0.16136
	NSGA-II	0.69862	0.29401		0.49282	0.17281
	MOEA/D	0.56320	0.24210		–	–
9	**MOSRS**	**0.99099**	0.18612	10	**0.32245**	0.25478
	MOGWO	0.83991	0.19759		0.29721	0.34651
	MOPSO	0.19816	0.16351		0.13015	0.06263
	NSGA-II	0.81573	0.15372		0.32138	0.01628
	MOEA/D	–	–		–	–

Regarding the IGD (Table 5), MOSRS had the best convergence mean in 5 of the 10 used test functions (represented in bold in Table 5), being the algorithm that won the most times. But it lost to NSGA-II in UF2; MOGWO in UF3 and UF5; and MOPSO in UF8 and UF9. Also, it is important to note that compared to the other algorithms, it has a good result in the three-objective test functions (UF8, UF9, and UF10), but MOPSO was the best, and this can be further investigated.

Regarding the mean distance between the Pareto front solutions measured by the MS metric and presented at Table 6, MOSRS had the best mean value just in UF5, showing that it is able to find more spaced solutions and with less continuity. The best algorithm in this metric was the MOPSO winning 4 times. But, connected with this, came the ability to find a well spread solution into the objective space. Very close solutions may indicate exploratory weakness.

Regarding the Maximum Spread, a measure between the furthest solutions in the PF, MOSRS had the best results in 8 of the tested functions, losing to MOGWO in UF3 and UF6. This is a significant achievement for our algorithm because it may suggest that it can present more options to the decision-maker when he analyzes the PF to make decisions.

**Fig 8:**
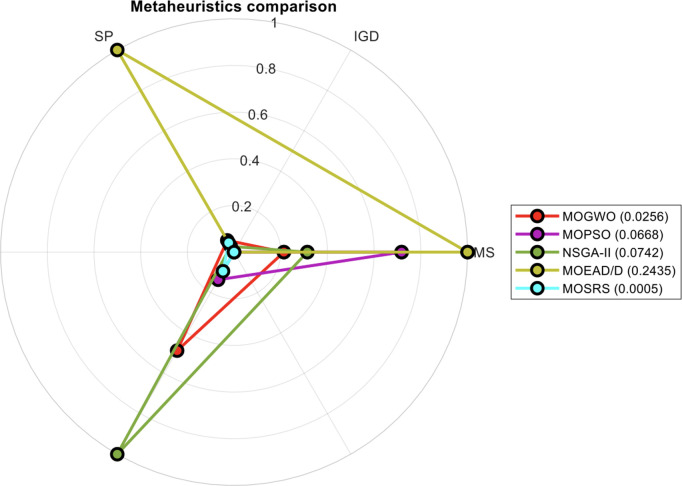
Summarizing the IGD, SP, and MS results with a radar plot.

The radar plot [45] in Fig 8 is a convenient tool to summarize the findings. It standardizes the key objectives of this study: IGD, SP, and MS. The area under the radar curve for each MH is indicated in parentheses. A smaller area reflects a more favorable balance among all the metrics and considers all test functions simultaneously. MOSRS achieved the smallest area (0.0005 – see legend in Figure), owing to its lowest IGD and SP. It is followed by MOGWO, MOPSO, NSGA-II, and MOEA/D. These results highlight the influence of computational cost on FS performance when it is considered.

**Fig 9:**
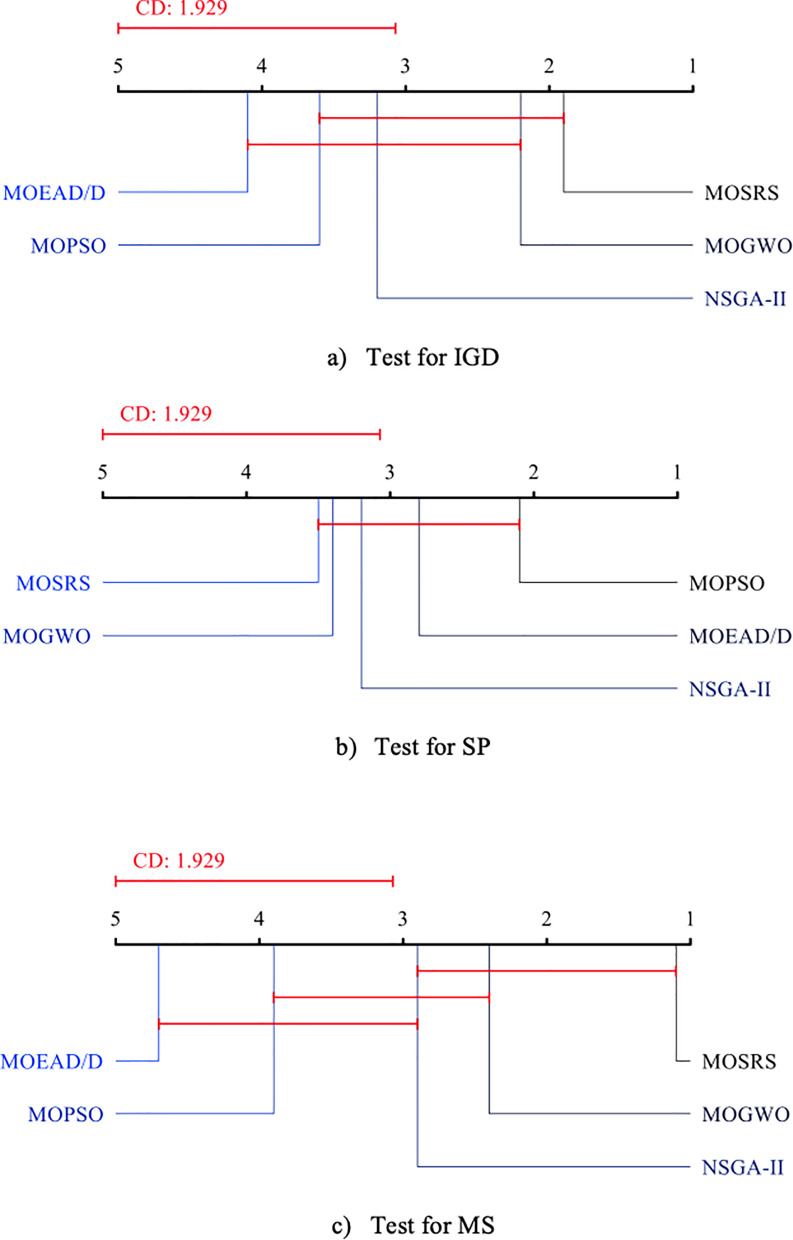
Friedman tests for the CEC 2009 problems.

To conclude the analysis of the algorithms’ performances on the CEC 2009 functions, the Friedman test [46] with 95% confidence is applied to the algorithms’ results for all test functions. They are shown in Fig 9 for the three metrics used. This test is commonly used to compare several machine learning algorithms on multiple problems. Regarding IGD, the test placed MOSRS as the best algorithm, followed by MOGWO and NSGA-II. MOEA/D and MOPSO are the worst algorithms.

Regarding SP, MOSRS is the worst, while MOPSO is the best. This metric measures how equally spaced the solutions are in the Pareto front. As for MS, MOSRS is again the best, and MOEA/D is the last. MOSRS was the best in the IGD and MS metrics, which measure respectively the convergence capacity and the total coverage of the Pareto fronts, revealing that it is an algorithm suitable for both.

**Fig 10:**
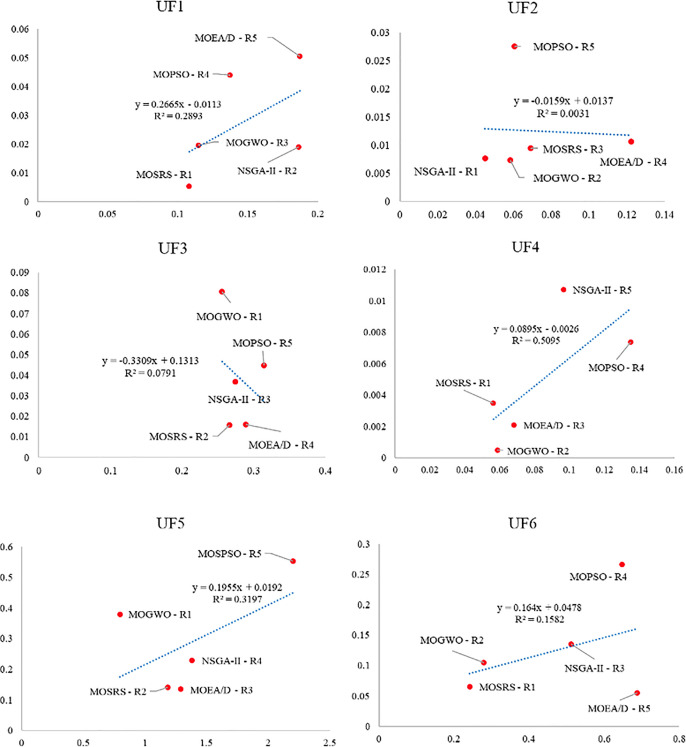
Graphical assessment of competitive algorithms by IGD metric (UF1:UF6).

**Fig 11:**
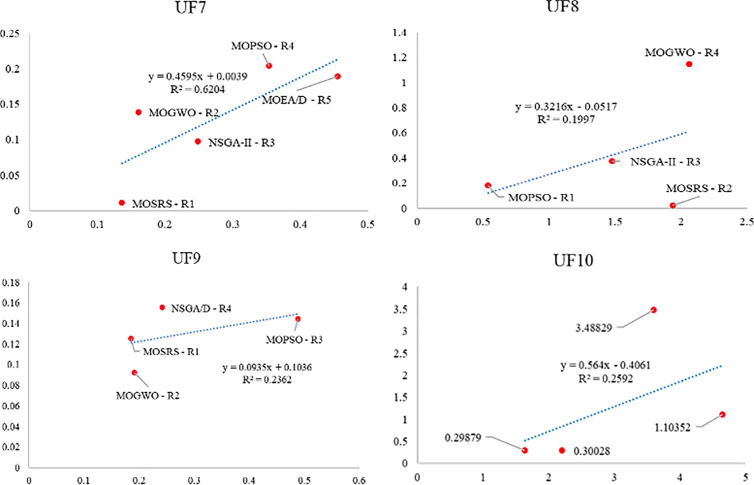
Graphical assessment of competitive algorithms by IGD metric (UF7:UF10).

**Fig 12:**
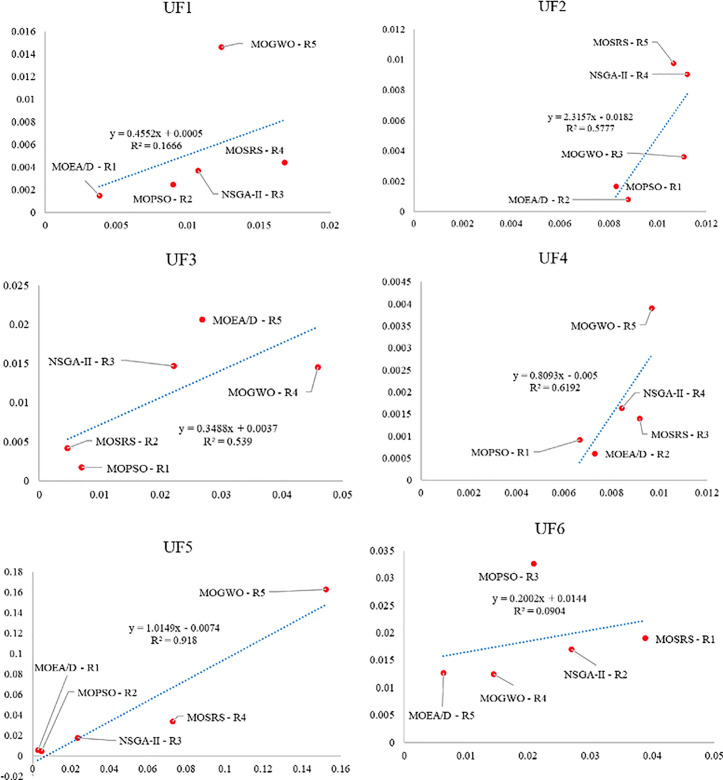
Graphical assessment of competitive algorithms by SP metric (UF1:UF6).

**Fig 13:**
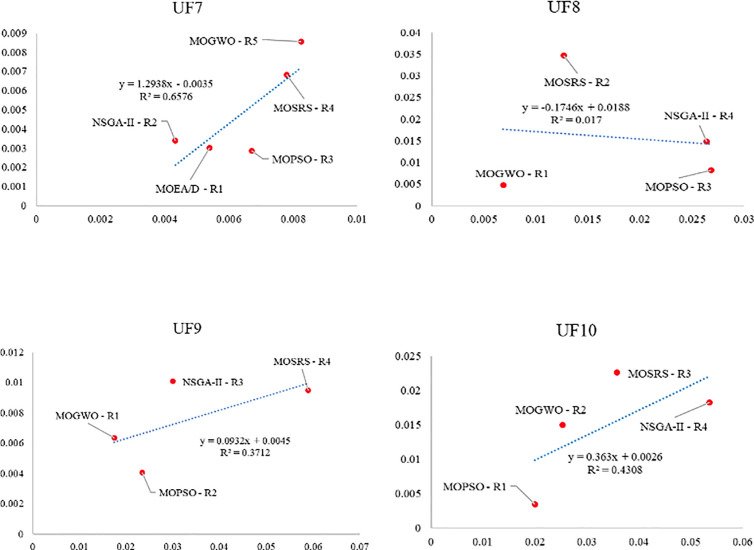
Graphical assessment of competitive algorithms by SP metric (UF7:UF10).

**Fig 14:**
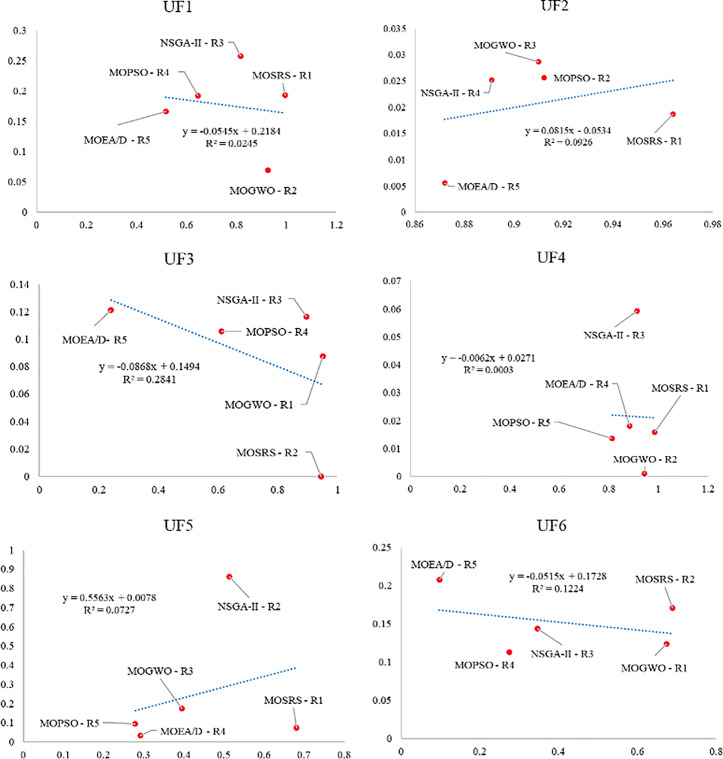
Graphical assessment of competitive algorithms by MS metric (UF1:UF6).

**Fig 15:**
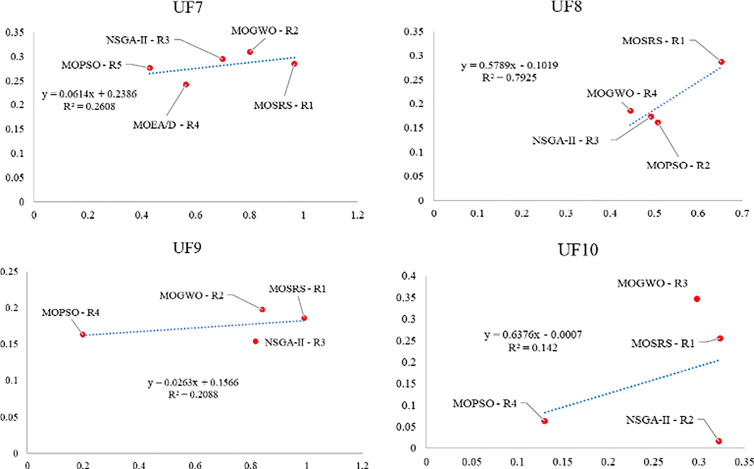
Graphical assessment of competitive algorithms by MS metric (UF7:UF10).

Also, Figs 10–15 analyze the performance of MOSRS, MOGWO, MOPSO, NSGA-II, and MOEAD regarding IGD, SP, and MS metrics. These figures represent trends in the data series, with linear equations calculated to illustrate the relationships. The algorithms are ranked based on their best performance from R1 to R5, where R denotes Rank. This ranking helps to identify the most effective algorithms for the given problems.

These figures show that the proposed method demonstrates superior overall performance in solving these problems. The trend analysis reveals that specific algorithms consistently outperform others across different metrics, highlighting their robustness and efficiency. Additionally, the lowest standard deviation (SD) of MOSRS is another critical metric, underscoring the reliability and stability of its results. This low variability indicates that MOSRS produces consistent outcomes, crucial for applications requiring dependable performance. Furthermore, the detailed analysis of these metrics provides insights into the strengths and weaknesses of each algorithm, allowing for a more informed selection process for specific problem-solving scenarios. The comprehensive evaluation and comparison enable researchers and practitioners to understand better the trade-offs involved in choosing an optimization algorithm and to tailor their approach to the specific needs of their application.

### 4.2. Design problems

**Fig 16:**
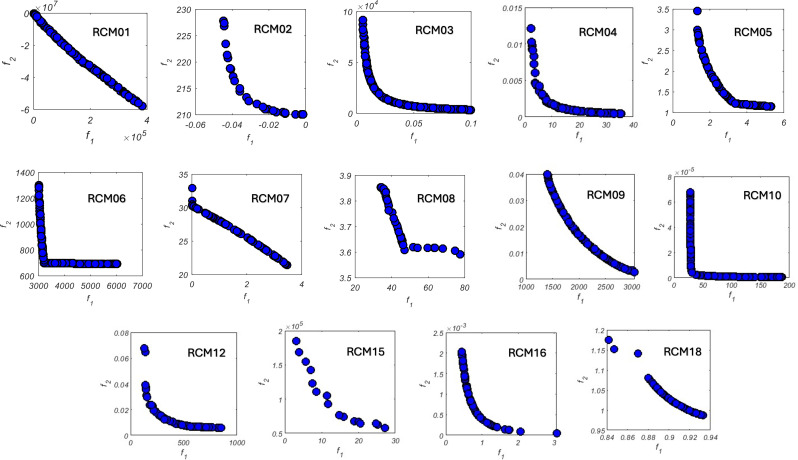
All bi-objective mechanical design optimization problems studied.

The mean results after 25 independent runs for all algorithms are presented in Table 8. The computer software used is the same. The MOSRS’s Pareto front of the bi-objective problems solved here are in Fig 16 for one of these runs. These problems do not have true Pareto Fronts; therefore, we will compare the algorithms using HV. The results are in Table 2, where it is possible to see that MOSRS showed results that were very competitive with the compared metaheuristics, having the best HV mean throughout all the problems with 0.3923 value. Following it comes AnD (0.3939) and NSGA-III (0.3897). The worst algorithm was TiGE2. Also, the metaheuristic that won more times was the ToP, having the best HV results on eight problems. Another interesting observation is about the AnD, which won just once but was consistent enough to have the second overall result.

**Table 8:** Mean HV.

Problem	MOSRS	ARMOEA	TiGE 2	NSGA-III	CCMO	ToP	MOSFO	AnD
RCM01	0.5764	**0.6070**	0.5110	0.6060	0.6040	0.6060	0.5921	0.5990
RCM02	**0.3813**	0.0360	0.0216	0.0535	0.0637	0.1500	0.1664	0.0384
RCM03	0.8511	**0.8980**	0.6880	0.8920	0.8970	0.7810	0.8925	0.8700
RCM04	0.8524	0.8530	0.4720	0.8540	0.8530	0.8610	**0.8620**	0.8530
RCM05	0.3438	0.4330	0.3970	0.4330	0.4330	**0.4340**	0.4287	0.4310
RCM06	0.2602	0.2770	0.2720	0.2770	0.2770	0.2740	**0.2772**	0.2770
RCM07	**0.4647**	0.2260	0.2000	0.2260	0.2270	0.2270	0.2283	0.2240
RCM08	**0.0265**	0.0259	0.0204	0.0254	0.0258	0.0256	0.0258	0.0258
RCM09	0.4088	**0.4100**	0.3230	0.4090	0.4090	0.4090	0.4084	0.4070
RCM10	**0.8571**	0.8410	0.8410	0.8330	0.8390	0.8470	0.8472	0.8450
RCM11	0.0949	0.0971	0.0979	**0.0997**	0.0992	0.0973	0.0933	0.0989
RCM12	0.6375	0.7220	0.6980	0.7220	0.7220	**0.7230**	0.5563	0.7180
RCM13	0.0864	**0.0903**	0.0867	0.0901	**0.0903**	0.0892	0.0879	**0.0903**
RCM14	0.5146	**0.6170**	0.3300	0.6160	**0.6170**	**0.6170**	0.6057	0.6060
RCM15	0.5215	0.5410	0.5090	0.5410	0.5410	**0.5430**	0.5427	0.5390
RCM16	0.7582	0.7620	0.7420	0.7620	0.7620	0.7630	**0.7641**	0.7590
RCM17	**0.2561**	0.2530	0.2040	0.2470	0.2530	**0.2650**	0.2592	0.2090
RCM18	0.0394	**0.0405**	0.0393	**0.0405**	**0.0405**	**0.0405**	0.0404	0.0404
RCM19	0.2773	0.2800	0.2780	**0.2850**	0.2800	**0.2850**	0.2066	0.2840
RCM20	0.1201	0.0031	0.0244	**0.1390**	0.0031	0.1090	0.0058	0.1160
RCM21	**0.0318**	0.0317	0.0212	0.0317	0.0317	**0.0318**	0.0317	0.0317
Avg	**0.3981**	0.3831	0.3227	0.3897	0.3842	0.3895	0.3772	0.3939

It is also possible to note that our method had the best results in one of the problems with more decision variables (RCM08), showing good abilities to handle high search space dimensional problems. However, when the number of objectives increases, such as in the RCM13 and RCM19, the performance of our proposed method decreases if compared with the other methods, which will be investigated more in the future. From Fig 16, we can see the MOSRS finding Pareto fronts with good coverage and convergence.

**Fig 17:**
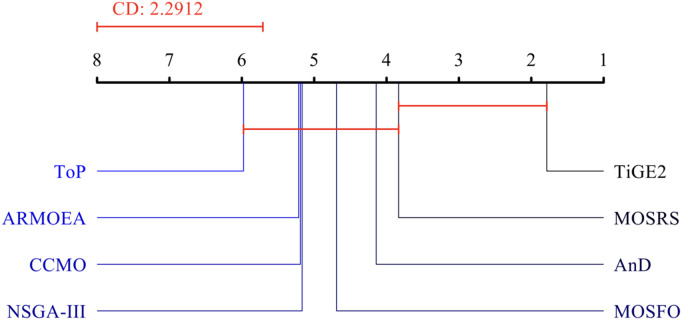
Friedman test for the Engineering problems.

As before, the Friedman test (See Fig 17) was used to compare these eight algorithms. TiGE2 was ranked as the best algorithm, and MOSRS was second. Behind it are AnD, MOSFO, NSGA-III, CCMO, ARMOEA, and ToP.

## 5. Conclusion

This paper presents the creation and validation of the Multi-objective Special Relativity Search, a multi-objective metaheuristic inspired by special relativity physics. This is the first time this theory has been applied to multi-objective optimization. MOSRS was validated using ten test functions from CEC 2009 being compared to NSGA-II, MOPSO, MOEA/D, and MOGWO in them and 21 complex structural design problems and compared with ARMOEA, TiGE 2, NSGA-III, CCMO, ToP, MOSFO, and AnD using the Hypervolume metric.

The Einsteinian approach had the best Inverted Generational Distance mean and Maximum Spread in the CEC 2009 test functions. It showed itself a promisor algorithm to explore the search space better and present Pareto fronts with more convergence and coverage. When applied to 21 real-world design problems, it had the best HV mean throughout all issues with a 0.3923 value. Following it comes AnD (0.3939) and NSGA-III (0.3897). The worst algorithm was TiGE2. It was possible to note that our method had the best results in one of the problems with more decision variables (7 in the Car Side Impact design), showing good abilities to handle high search space dimensional problems. However, when the number of objectives increases, such as in the Gear Box Designs (7) and Multi-Product Batch Plant (10), the performance of our proposed method decreases if compared with the other techniques, which will be investigated in the future, this was also noticed in the CEC2009 test problems.

MOSRS obtained good coverage and convergence capabilities and proved very competitive with the compared metaheuristics, which were selected to be common, recent, and popular. The algorithm was shown to be very promising with higher decision variable dimensions and was specially tested for real-world structural design problems. While the proposed MOSRS algorithm has demonstrated strong performance across benchmark and real-world problems, future work will extend its capabilities to handle many-objective problems (more than three objectives) more effectively. Additionally, we aim to explore adaptive mechanisms for further enhancing convergence and diversity, as well as integrating hybrid approaches and applying MOSRS to emerging complex engineering applications such as smart structures and energy systems.
